# Informal work and formal plans: articulating the active role of patients in cancer trajectories

**DOI:** 10.5334/ijic.822

**Published:** 2012-12-18

**Authors:** Rikke Juul Dalsted, Bibi Hølge-Hazelton, Marius Brostrøm Kousgaard, John Sahl Andersen

**Affiliations:** The Research Unit for General Practice and Section of General Practice, Department of Public Health, University of Copenhagen, Copenhagen, Denmark; The Research Unit for General Practice and Section of General Practice, Department of Public Health, University of Copenhagen, Denmark; The Research Unit for General Practice and Section of General Practice, Department of Public Health, University of Copenhagen, Denmark; Section of General Practice and Research Unit for General Practice, Department of Public Health, University of Copenhagen, Copenhagen, Denmark

**Keywords:** patient role, patient trajectory, pathway models, cancer

## Abstract

**Introduction:**

Formal pathways models outline that patients should receive information in order to experience a coherent journey but do not describe an active role for patients or their relatives. The aim of this is paper is to articulate and discuss the active role of patients during their cancer trajectories.

**Methods and theory:**

An in-depth case study of patient trajectories at a Danish hospital and surrounding municipality using individual interviews with patients. Theory about trajectory and work by Strauss was included.

**Results:**

Patients continuously took initiatives to organize their treatment and care. They initiated processes in the trajectories, and acquired information, which they used to form their trajectories. Patients presented problems to the healthcare professionals in order to get proper help when needed.

**Discussion:**

Work done by patients was invisible and not perceived as work. The patients’ requests were not sufficiently supported in the professional organisation of work or formal planning. Patients’ insertion and use of information in their trajectories challenged professional views and working processes. And the design of the formal pathway models limits the patients’ active participation. When looking at integrated care from the perspective of patients, the development of a more holistic and personalized approach is needed.

## Introduction

Today’s healthcare systems are complex with a large number of professional actors providing health services across multiple organisational settings [1–5]. In such systems, the roles and responsibilities of the different providers in specific situations are sometimes unclear [6], which can result in fragmented patient care where the necessary mix of services is not delivered sequentially or simultaneously when needed [7]. In response to the increasing specialisation and fragmentation of healthcare provision [2–5], managerial inspired ‘pathway models’ have been introduced in several countries to ensure efficient and integrated patient care [8–11]. In Denmark, the national integrated cancer pathways focus on the “the journey of the patient through the healthcare system” with the official objective of reducing processing times and promoting standardised treatment and care of high-quality [12]. These national guidelines stipulate the tests, treatments and time standards that are required for the various steps in the process, from referral through examination and treatment to follow-up.

Although the formal pathway models outline that patients should receive information about examinations and treatments in order to experience a cohesive trajectory, the pathway models do not describe a particularly active or inclusive role for either patients or their relatives. However, studies have shown that chronically ill patients perform various kinds of work during their time in hospital and after discharge [13–15], and that many patients would like an active role in managing issues related to their cancer treatment [16]. Even so, few studies have explored in detail what patients do to manage their trajectories while various health organizations are providing their treatment [Ibid.]. Furthermore, the lack of knowledge in this area limits the possibilities for involving the perspectives of the patients in the planning and evaluation of health care services [17].

Conceptualizing the patients’ actions in terms of work serves to highlight and recognize the active efforts of patients—efforts that are not always visible from an administrative or professional perspective [5, 14]. The term ‘work’ furthermore avoids the connotation (indicated by ‘participation’) that the healthcare professionals are working on the main trajectory while the patients are taking care of secondary aspects of their trajectories [14]. This focus on patient work is aligned with an understanding of patient trajectories (or ‘illness trajectories’) as referring not only to the physiological unfolding of a patient’s illness, but also to the total organisation of work done over the course of an illness, plus its impact on those involved with that work and its overall organisation. This includes treatment and care, as well as other matters that arise as tasks to be done [18]. Thus, the notion of trajectory allows for a comprehensive view of cancer treatment and care that includes the patients’ actions in a context where healthcare is provided by a multiplicity of healthcare professionals and organisations. In this paper the aim is to articulate the active role of patients in shaping their trajectories during treatment for cancer in order to avoid fragmentation and increase coherence. More specifically, the paper explores self-initiated patient work.

## Method and design

### Design

This paper is a part of a larger in-depth case study [19] of collaboration (inter professional as well as between professionals and patients) in patient trajectories in cancer treatment and care. The study was mainly explorative and began with a few core themes about collaboration. The setting was a Danish hospital and the cooperating healthcare providers in the local municipality. We chose an information-rich case [20] that was representative of the Danish hospital setting in the way that the treatment and care was organised among different hospitals, hospital wards and healthcare providers in the surrounding municipality. The hospital at which the patients were recruited was their primary hospital (i.e. it was responsible for diagnosing their diseases and planning treatments), and the patients often returned there after special or additional treatments at other hospitals.

Interviews with 12 patients diagnosed with cancer were conducted between autumn 2007 and autumn 2008. The patients were interviewed using semi-structured research interviews [21, 22]. In order to articulate and understand the patients’ perspectives and experiences, the patients were asked to describe their trajectories and reflect on their experiences and encounters with healthcare professionals. The interviews focused on the period from when the patient first contacted his/her general practitioner (GP) due to symptoms until the end of treatment. The selection criteria for patients were:

- Diagnosed with colorectal, pulmonary or prostate cancer (these are the most frequent cancers in Denmark along with breast cancer, which is not included because special pathways have been developed).
- In treatment or recently finished treatment.
- Had contact with at least two hospital wards during diagnosis and treatment.

With consideration to the above criteria, the patients were recruited by the responsible physician or contact nurse at the hospital. If a patient was interested in participating, he/she was given a letter of invitation we had written, which informed the patient about the study and his/her possible contribution. After this, we phoned the patients to arrange a date for an interview that could take place in their homes, at the hospital or at the university. All the patients chose their own homes, and in the majority of cases, their spouses participated in the interviews. Some spouses only provided brief comments, while others contributed more with their own descriptions of the patient’s trajectory. This inclusion of the spouses was not planned by us beforehand, but happened on the initiative of the patients and/or their spouses.

The interviews were conducted by two persons with extensive experience with the method (one of them the first author of this article). During the interviews we asked the patients to describe how their trajectory had unfolded from the first symptom to the end of treatment. The interviews also included questions about which healthcare professionals the patients had contacted when in need of help, how they had collaborated with their GP and healthcare professionals at the hospital, and how the patients experienced this collaboration in terms of positive and negative aspects. As part of the interview the patients described what they themselves had been taking care of during the treatment. Results from previous research and literature were used to select the themes for the interview guide [23–27]. The interviews each took 1–2 h and were recorded and transcribed verbatim. An overview of the participating patients is shown in Table 1.

The analysis of the interviews was done as a process with systematic text condensation inspired by Giorgi [28, 29] and meaning condensation [21] done by two persons independently reaching consensus. The analysis was primarily data-driven [29; 97] and was performed in 4 steps:

1. The first step of the analysis was reading through all the empirical data and creating overall themes that were recurrent throughout the data and represented aspects of collaboration between the patients and health care professionals. The themes were partly based on what was mentioned in the interview guides.
2. The next step was to organise the part of the data that we wanted to investigate further, and sorting out the text that could help clarify the research questions. This was done by creating meaning-bearing codes [29]. The codes were developed when reading through the data in detail about the themes in step one. (For codes see Table 2.)
3. In the third step the codes were divided into groups. Steps 2 and 3 were closely connected.
4. The final step was to relate the developed themes and codes from the data to relevant research on collaboration between the various actors in integrated care [14, 30].

In the process some of the themes and codes were discontinued and some were revised as the analysis unfolded. Table 2 gives an overview of the steps of analysis and the objectives of each level.

### Ethics

The project was declared to the Danish Data Protection Agency, and the research was performed according to the standards of the Danish Research Ethics Committee and the Declaration of Helsinki.

## Findings

The analysis revealed that patients and their relatives performed numerous kinds of work in order to prevent interruptions and delays, and to ensure their own comfort during the trajectories. There were no differences in the level of patient activity across the different types of cancer. Below we present a list of examples of how the patients actively shaped their trajectories:

- In order to obtain an overview of their treatment process, the patients often used a calendar or notebook to keep track of appointments, test results or other aspects of their treatments that they found important to remember or difficult to keep track of. They sometimes used this information to remind the healthcare professionals about planned examinations and tests. When they acquired information about their illnesses, treatments, side effects or legal rights, the patients often used this information to influence their trajectories. For example, acquiring information about the legal time limits for examination scanning for diagnosis, and then using this knowledge to get scanning done at another hospital.
- When they were asked about it or when the patients perceived it to be important, they provided information to professionals in one setting about what happened during treatment in another setting. For instance, a patient provided information to one team in the hospital ward about a meeting with another team at the outpatient clinic.
- Actively engaged in decisions over what medications to use and when some patients reduced or stopped taking their medication when they experienced side effects (e.g. morphine as a painkiller) or deemed the medication to be unnecessary. Others asked for specific medication to be prescribed (e.g. a certain brand of medicine or calcium/vitamin D).
- Suggested and received a control scanning during treatment at the hospital, e.g. when a scanning was not part of the treatment plan, and the patient was anxious to know if the treatment had any effect.
- Took initiative to get a scanning done at a private hospital due to long waiting times at the planned hospital. The images were subsequently included in the trajectories.
- Organised overnight stays at patient hotels or hospital wards in order to reduce transportation time and avoid the discomfort involved with this (for instance, when the patients had treatment scheduled the next day at the same hospital).
- Arranged for care materials to be provided in their homes to ensure the continuation of care after discharge. For example, a patient ordered materials for ostomy care from a company and had them sent to a nearby nursing home where the patient could pick them up. The local municipality had originally planned for the provision of these materials, but the process failed.
- Initiated treatment that was otherwise not planned. For instance, a patient who had witnessed a progression in the disease had his case reopened, not accepting that no further treatment was planned.

Apart from these kinds of actions, a significant aspect of the patients’ active role was to present emerging problems to the healthcare professionals that the patients needed help solving. The majority of patients emphasized the importance of being provided with help in such situations and often highlighted specific events in the trajectories; they labelled these as either good or bad, depending on the response or help they received when presenting a problem. When the problems presented fit into the flow of the professionals’ organisation of work, they were usually taken care of by the professionals. However, when the problems presented did not fit the professionals’ organisation of work, the patients often experienced that they did not receive a satisfactory response. For example, due to medical specialisation, treatment was sometimes partially distributed between hospitals, in a way that could result in long and uncomfortable transportation from a patient’s home to the hospital or between the hospitals, even in situations of physical and/or mental distress. But such unintended discomfort was not always responded to when expressed by the patient. In such cases the patients were frustrated and confused about why they were asked to go somewhere else. The patients did not perceive their situation as being split according to the formal division of responsibility between health professionals. When they perceived a problem, the patients contacted a professional whom they knew and trusted, with no regard to formal areas of responsibility. For example, a doctor told a patient diagnosed with prostate cancer that he did not need treatment, but the patient did not accept this. Therefore, he contacted two nurses he knew from the ward in order to get his case reopened:

Patient: I did not like the message I got the first time that ‘we’ll just keep an eye on you every three months’.

Interviewer: So what did you do? Did you contact the doctor, your GP or the outpatient clinic?

Patient: No, I called the two nurses at the ward who could help me with an answer that the doctor could not... They knew.

The analysis showed that the patients were aware of their own active role in the trajectories. They were conscious that their particular trajectory was part of a large overall system and planning, and they did not complain about having to contribute:

Patient A: They (the professionals) take care of a lot of people, and I do not think they have time for each person.

The patient did not see it as a problem that his trajectory was part of the healthcare system as a whole and he needed to participate actively. Rather, most of the patients perceived themselves as part of a team and as partners with the health professionals:

Patient B: In my case, it was a team. It was us doing something together... And on top of that, we succeeded (in curing the cancer).

In addition to the healthcare professionals, the patients’ relatives were their most significant partners. Many patients emphasised the importance of having a relative to support them with both practical and psychological matters. Two patients expressed it this way:

Patient A:

Interviewer: I’m thinking about the patients who do not have a relative like John (pseudonym) next to them. What do they do?

Patient A: I have thought about that many times... I don’t know what I would have done without him, to be honest.

Patient B:

Relative: Yes, I am the secretary.

Interviewer (to the patient: What if you did not have a secretary like that?

Patient B: It is really important... My wife here is a nurse assistant, so she knows a bit about it, right? So that is a plus.

The importance of relatives was also obvious at the interview sessions, where 10 out of the 12 patients had their spouses participating in the interview. Of the remaining two patients, one was himself a supporting spouse (having a wife with dementia), while the other had support from her sister, who was not present at the interview.

During the interviews the patients were asked why they and their relatives sometimes chose to handle problematic issues by themselves. Most of the patients replied that they had not actually considered asking for help when they had come up with solutions themselves. Also, when they did ask for assistance and the professionals were not able to provide the help they needed, the patients and their relatives would attempt to find a solution themselves.

## Discussion

The focus of formal pathway models tends to be on organisational boundaries and medical guidelines specifying the actions and responsibilities of health professionals [18]. In contrast, we have focused on the actions taken by patients during their trajectories—in this context understood and conceptualized as work [14–15]. The study showed that patients (and/or their relatives) continuously took initiatives to organise their treatment and care. They initiated specific processes in their trajectories, and acquired information about their illnesses and situations that they used to form their trajectories. Also, the patients presented problems to the healthcare professionals during their trajectories in order to get proper help when needed. These various kinds of work carried out by the patients during the treatment for cancer seem to suggest that a number of ‘non-formalized tasks’ and issues perceived as important by the patients are not being addressed by the formal health care system. Thus, the patients’ actions often functioned as ‘glue’ in the trajectories, and among other things helped to manage the transition from one provider to another. So although patients are not formally assigned work, the ongoing work performed by patients in the study shows that formal planning is not the only mechanism for connecting tasks and creating coherency within the trajectories. And while formal pathway models are prospective and create an explicit agenda for the patient trajectories, the actual patient trajectories might turn out differently and include several situations and events not accounted for in the formal planning of generic and specific trajectories. In such situations, problems may arise for the patients, who may respond in different ways—sometimes by dealing with the problem themselves and sometimes by presenting the problems to the professionals. For example, part of the patients’ work in the study was to acquire information about their diagnoses, treatments and legal rights from a wide range of sources, such as the Internet, patient organisations, TV programmes, magazines, etc. The patients actively used this information when asking for professional help and organising their trajectories e.g. when they acquired information about the maximum waiting time for a scanning allowed by law, or when they independently organised the delivery of materials to care for their ostomies. However, several patients in our study had experienced that such presenting of problems were not always sufficiently handled by the professionals, and the lack of response to these problems was a major source of frustration and dissatisfaction for the patients.

The patients’ experiences of presenting information and problems that were not adequately responded to may have several causes: First, health professionals can have an inclination to rely on information that has been provided by other health professionals [14], entailing that patients’ views about their own situations are not considered to be of equal importance. Thus, the patients’ insertion of information to handle or draw attention to perceived problems may not always be perceived as legitimate or appropriate in a medical field where valid information tends to be construed as expert knowledge [31]. Contrary, patients generally do not care where the information comes from, only whether it is helpful to them on a daily basis [Ibid.]. Second, although the work of patients moves healthcare work away from the medical sphere, and binds the private sphere of the patients’ lives closer to the medical sphere of the professionals [15, 32], the professionals’ approach does not always consider the multidimensionality and the specific contexts of the patient’s lives. From the perspective of a patient seeking help, medical and social needs may be closely related. The patients do not see themselves as ‘multi-ill’, but rather as needing help with their problems as they know them [5]. These considerations lead to a related explanation, namely that the problems (and possible solutions) presented by patients do not always fit the professional organisation of work, as in the case of patient transportation mentioned in the previous section. This tendency may be augmented by the fact that the primary focus of the pathway models is on the technical and medical dimensions, while patient involvement and two-way communication is not thematized. Although the Danish pathway models for cancer [12] include guidelines for the written and verbal information that is given to patients, the design of the models limits the patients’ active participation because this communication is designed to move in one direction from healthcare professional to patient, in order to ensure that the patient are well-informed [33–35]. Furthermore, the role of relatives is not included in the models. Patients are often encouraged by healthcare professionals to bring a relative to meetings and examinations, but the patients and their relatives are not represented as resourceful actors in the pathways.

These observations point to the uneasy relationship between two major logics in modern health care systems 1) the logic of providing standardized health care services for all patients (e.g. the formal pathway models) [10], and 2) the logic of patient involvement. The purpose of having standardized models is to secure coherent treatment and care across organisational boundaries in such a way that one organisational part of the system can arrange the next step in the patient trajectories and both parts know what to expect from each other. On the other hand, the logic of patient involvement emphasizes that services should be provided with a consideration for the specific needs, viewpoint and situation of the individual patient. It is difficult to fully integrate these two logics simultaneously in the formal planning and execution of patient trajectories, and recent studies suggest that the actual involvement of patients is still very limited or non-existent, owing to the way healthcare provision is organised [35, 36].

### Limitations of the study

Our study involved a detailed investigation of cancer patients’ trajectories in connection with a regional hospital and municipality in Denmark. With regards to the transferability [30] of the results, one must be aware that the work and experiences of patients may be different in other settings, depending on the organisation of the trajectories. For example, in the present case, examinations and treatments took place at various hospitals (owing to the formal organisation of dividing the treatment between smaller hospitals), and this increased the amount of travelling done by the patients, which resulted in some frustrating experiences. At a larger city hospital, the need to travel between hospitals might be reduced, and thus the work and experiences of the patients would be different in these more centralised settings.

Moreover, the selection and number of patients included in the study was influenced by the focus of the overall study, and the specific process of selection employed *may* have introduced a bias towards particularly active patients. The significance of the socio-economic status of patients for the types and amount of work carried out could be be an issue for research.

Finally, our study was explorative and focused on the work of patients to increase coherency and avoiding fragmentation. However, there are several other types of work that patients engage in at home and in the clinical settings (e.g. psychological work, monitoring work, body work etc.) [13–15, 31] and future research could systematically investigate the relationships and implications of the different kinds of work that patients and relatives perform during patient trajectories.

## Conclusion

Formal models of integrated care, based on principles of standardisation, have been introduced in the Danish healthcare system as an answer to problems of coordination and fragmentation, and this trend has been especially profound within cancer treatment. At the same time, ideas about patients’ resources, participation and potential contributions to the processes and outcomes in healthcare have increasingly received policy attention [15]. As this study has shown, patients and/or their relatives perform a wide range of actions to shape their trajectories in and across various healthcare settings. Yet, this work is not represented in formal pathway models for cancer treatment, and it is not always recognised by healthcare professionals. As Kodner and Spreeuwenberg point out [7], a lack of integration of patients’ perspectives and contributions in healthcare affects both professionals and patients, but not equally. Thus, when looking at integrated care from the patients’ perspectives, a more holistic and personalised approach needs to be developed. However, the nature and scope of patient work varies significantly among individual patients. This makes patient work extremely difficult to inscribe in formal plans, and it seems to constitute a limitation in the capacity of formal pathway guidelines to create cohesive trajectories from the perspective of an individual patient. Nevertheless, contemporary discussions about integrating patient resources and perspectives should take more explicit account of the work that patients (and relatives) are already performing, and consider how such work (and the needs and experiences that motivate it) relates to current models for standardised treatment and care. Also, while this study highlights the active role of patients, it should be emphasised that not all patients have the same resources, nor are they able to (or wish to) perform the same amount of work. The future development of pathway models and other formal interventions to facilitate integrated cancer treatment and care should explore how to simultaneously manage standardisation and specialisation as well as the individual work and resources of patients, and the obligation of healthcare professionals to attend to their individual needs.

## Reviewers

**Carina Berterö,** Professor in Nursing Science, Linköping University, Sweden

**Hannele Kerosuo**, PhD, University Researcher, Centre for Research on Activity, Development and Learning, Institute of Behavioural Sciences, University of Helsinki, Finland

One anonymous reviewer.

## Figures and Tables

**Table 1.** Overview of the participating patients

Patient	Sex	Age in years	Spouse/Relative participating in interview	Type of cancer
1	Male	73	Yes	Lung
2	Female	46	No (but had a sister as support)	Lung
3	Female	60	Yes	Lung
4	Male	68	Yes	Lung
5	Male	69	Yes	Lung
6	Male	77	Yes	Lung
7	Female	65	Yes	Rectal
8	Male	54	Yes	Rectal
9	Male	70	Yes	Colon
10	Male	60s	Yes	Colon
11	Male	63	No (his wife was very ill)	Prostate
12	Male	72	Yes	Prostate

**Table 2.**
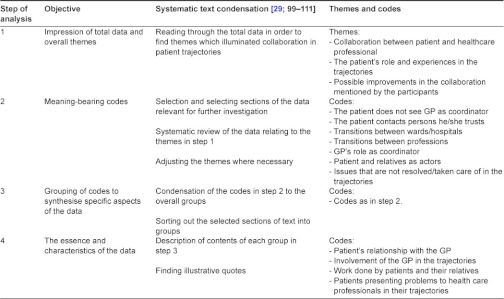
Overview of steps of analysis
